# Identification and Expression Analysis of a Novel HbCIPK2-Interacting Ferredoxin from Halophyte H. *brevisubulatum*

**DOI:** 10.1371/journal.pone.0144132

**Published:** 2015-12-04

**Authors:** Chao Zhang, Rongchao Ge, Junwen Zhang, Yajuan Chen, Hongzhi Wang, Jianhua Wei, Ruifen Li

**Affiliations:** 1 Beijing Key Laboratory of Agricultural Genetic Resources and Biotechnology, Beijing Agro-biotechnology Research Center, Beijing Academy of Agriculture and Forestry Sciences, Beijing, China; 2 College of Life Science, Hebei Normal University, Shijiazhuang, China; 3 Department of Biochemistry and Molecular Biology, Institute of Basic Medical Sciences, Chinese Academy of Medical Sciences & Peking Union Medical College, Beijing, 100005, China; University of Western Sydney, AUSTRALIA

## Abstract

Ferredoxin is a small iron-sulfer protein involved in various one-eletron transfer pathways. Little is known about how ferredoxin is regulated to distribute electron under abiotic stress. Our previous study has showed that HbCIPK2 conferred salinity and drought tolerance. Thus, we hypothesized that HbCIPK2 could mediate the activities of interacting partners as a signal transducer. In this report, we identified a novel HbCIPK2-interacting ferredoxin (HbFd1) from halophyte *Hordeum brevisubulatum* by yeast two-hybrid screens, confirmed this interaction by BiFC in vivo and CoIP in vitro, and presented the expression pattern of HbFd1. *HbFd1* was down-regulated under salinity and cold stress but up-regulated under PEG stress, its expression showed tissue-specific, mainly in shoot chloroplast, belonging to leaf-type subgroup. Moreover, HbCIPK2 could recruit HbFd1 to the nucleus for their interaction. The C-terminal segment in HbFd1 protein was involved in the interaction with HbCIPK2. These results provided insight into the connection between CBL-CIPK signaling network and Fd-dependent metabolic pathways.

## Introduction

Chloroplast ferredoxin (Fd) is an important electron transfer protein in photosynthetic organisms. Fd plays a pivatol role in plant cell metabolism, in addition to the primary function in photosynthesis, it works not only in many essential metabolic reactions such as biosynthesis of chlorophyll, phytochrome and fatty acids, assimilation of sulphur and nitrogen, but also in redox signaling [1].

Recently, overexpression of Fd in plant was found to enhance tolerance to abiotic and biotic stress [2, 3]. It was because that Fd could down-regulate reactive oxygen species (ROS) level produced under adverse environments through the ascorbate-mediated water-water cycle, a ROS-scavenging pathway [4]. However, the level of Fd transcripts and protein has been observed to decrease under drought, cold, or salt stress in some plants [5]. Whatever, light- and stress-dependent regulation of Fd expression may proceed through different pathways, but it is not clear if there is a switch between light-dependent induction and stress-dependent repression for Fd expression process.

It is vital for Fd to transfer eletrons to a variety of corresponding enzymes via specific protein-protein interaction [6]. However, recent researches mainly focused on the interaction of Fd with downstream proteins that act as Fd-dependent metabolic reactions and the interaction sites [7, 8]. So far, there have been no reports on which regulatory proteins interact with Fds to mediate them.

Calcineurin B-Like-interacting protein kinase (CIPK) is a kind of plant-specific regulatory protein which interacts with calcineurin B-like (CBL) to form complex, then as a signal transducer CIPK interacts with downstream protein such as SOS1 [9], AKT1 [10] or RBOHF [11] to phosphorylate them, at last regulate their functions. To date, many components of CBL-CIPK signaling pathways have been identified and functions of partial CBL-CIPK system responsive to abiotic stress also have been dissected [12]. At the same time emerging reports indicate few specific CBLs or CIPKs function in plant developmental regulation [13, 14]. Although CIPKs have been found to interact with diverse and broad range of protein targets, the role of CBL-CIPK in plant metabolism still requires further investigation. HbCIPK2 was identified by cDNA-AFLP technique from halophyte *H*. *brevisubulatum*, overexpression of it in *Arabidopsis* could enhance tolerance to salt and drought [15]. But the interacting partners of HbCIPK2 have not been identified. In addition to partners involved in response to abiotic stress, other target proteins participated in cellular metabolism will be our aim because they may connect some metabolic pathways. As for *H*. *brevisubulatum*, it is one of wild relatives of cultivated barley, called as wild barley, and belongs to a kind of halophyte that has evolved for long time to thrive in saline condition with strong tolerance to salinity and drought [16]. Thus it is important to investigate components of the HbCIPK2 interactome in wild barley.

In this report, we identified the interaction partners using HbCIPK2 as bait in yeast two-hybrid screens, and confirmed the interaction between HbCIPK2 and HbFd1 by cellular bimolecular fluorescence complementation (BiFC) and biochemical co-immunoprecipitation (CoIP) assays. Moreover, HbCIPK2 can recruit HbFd1 to the nucleus for their interaction, the key interaction segment of HbFd1 with HbCIPK2 has been investigated, and the expression pattern of HbFd1 also was presented. These results suggested the connection between the CBL-CIPK system and Fd-dependent metabolic pathways.

## Materials and Methods

### Plant materials and growth conditions

Collection of seeds of *H*. *brevisubulatum* from salinized grassland in Inner Mongolia of China is mainly used for scientific study, no specific permission is required. We confirmed that this field collection did not involve endangered or protected species. For the construction of yeast-two hybrid cDNA library, gene cloning and expression analysis, seeds of *H*. *brevusubulatum* (Trin.) Link were used and collected here. Seedlings were grown in half-strength Hoagland nutrient solution at 22°C with a 16 h light/8 h dark photoperiod and an irradiance of 120 μmol m^-2^ s^-1^. For the BiFC, subcellular localization and western blot analysis, the *Arabidopsis* wild-type (ecotype Columbia) was used and sown in potting soil mixture (rich soil:vermiculite = 2:1, v/v) under a 14/10-h light/dark cycle at 21–22°C with an irradiance of 120 μmol m^-2^ s^-1^. The relative humidity was approximate 70% (±5%).

### Yeast two-hybrid cDNA library screening and pairwise assay

A yeast two-hybrid cDNA library was constructed from the mixed seedlings of *H*. *brevisubulatum* treated with 430 mM NaCl and 350 mM mannitol for 30 min, respectively, upon the emergence of the third shoot according to the manufacturer’s manual of CloneMiner II kit (Invitrogen, Carlsbad, CA, USA) by Invitrogen company. The plants were cultured using the method described by Li et al. [15]. The activation domain (AD) expression vector contained a cDNA library with colony-forming units (CFU) of 0.552×10^7^, the recombination rate is 94%, and the average insert size is greater than 1kb in identified library, these matched the requirements of the library screening. The *HbCIPK2* coding sequence was cloned into the pDEST32 which is GAL4 binding domain (BD) expression vector by gateway technology. Using HbCIPK2 as a bait, the cDNA library was screened by following the manufacturer’s instructions for ProQuest™ Two-Hybrid system (Invitrogen, USA). Approximately 1.8×10^6^ yeast transformants were screened on the selective medium (SC/-Leu-Trp-His) with 35 mM 3-amino-1,2,4-triazole (3-AT). The β-galactosidase activity was determined in yeast strain MaV203, and the positive candidates were subjected to sequencing. After sequencing, the coding sequences (CDS) of selected inserts were amplified with advantage HD polymerase (Clontech Laboratories, Inc., CA, USA) and cloned into prey vector pDEST22 again, the fusion prey and HbCIPK2 bait vector were co-transformed into yeast strain Mav203 (*MATa*, *HIS3*, *lacZ*, *trp1*,*leu2*,*and ura3*), the other manipulations were performed according to the manual above (Invitrogen). For yeast-two hybrid pairwise assay, the indicated bait vector and prey construct were transformed into the yeast Mav203 strain, and 10-fold dilutions (A_600_ of 1–10^−4^) of yeast clones were spotted on selection media without Leu and Trp (selection for positive transformants) or without Leu, Trp, His and Ura (with 35 mM 3-amino-1,2,4-triazole; selection for interaction) and incubated for 3–5 days at 30°C. The interaction of positive clones was confirmed by ß-Gal assays. The plasmids provided by Invitrogen system were used as positive and negative control to monitor the conditions in this assay.

### Bioinformatics analysis of HbFd1

A PSI-BLAST search for HbFd1 homologs was performed using the NCBI BLAST server. Multiple sequence alignment was performed using the program ClustalX1.83. Conserved domains of HbFd1 were predicted using the ChloroP server (http://www.cbs.dtu.dk/services/ChloroP/) and the National Center for Biotechnology Information (NCBI) Conserved Domains database (http://www.ncbi.nlm.nih.gov/Structure/cdd/wrpsb.cgi). Peptides of active center of HbFd1 were aligned with that of six barley Fd proteins using DNAman software. The phylogenetic tree was generated with the amino acid sequences of HbFd1 and other plant Fd proteins using MEGA4 with 1000 bootstrap replicates. After assembly of *HbFd1* cDNA, it was amplified from cDNA and genomic DNA of *H*. *brevisubulatum* using the specific primer pairs listed in S1 Table. The PCR conditions were as follows: an initial denaturation at 94°C for 3 min, followed by 30 cycles at 94°C for 30 s, 58°C for 30 s, 72°C for 1 min, and a final extension at 72°C for 10 min. Sequence analysis was used to confirm if *HbFd1* has intron in genome.

### Transcript of *HbFd1* by real-time PCR

To confirm expression of *HbFd1*, real-time PCR (RT-RCR) was performed in an optical 96-well plate with SYBR Premix Ex Taq (TaKaRa BIO INC., Otsu, Shiga, Japan) using a Bio-Rad CFX96 real-time system. Total RNA from *H*. *brevisubulatum* seedling treated with various stresses (350 mM NaCl, 350 mM mannitol and 10% PEG6000 stressed for 6 hrs, and 4°C for 12 hrs, respectively) upon the emergence of the third shoot was extracted using RNeasy Plant Mini kit (QIAGEN, Stockach, Germany) and first strand cDNA was synthesized using the SuperScript III First-Strand (Invitrogen). The specific primers for *HbFd* and *GAPDH* as a control were listed in S1 Table. Some RNA from different tissues (young root, young leaf, steam, young inflorescence, leaf sheath, mature leaf, mature root and anther) was used to evaluate the spatial and temporal expression of *HbFd1*. The PCR conditions were 95°C for 15 min followed by 40 cycles at 95°C for 10 s and 60°C for 30 s. All reactions were performed in triplicate, and each reaction contained 10 μl SYBR Premix Ex Taq, 3 μl cDNA, and 10 μM specific primers in a final volume of 20 μl. The specificity of the amplifications was first confirmed by the presence of a single band of expected size for each primer pair in agarose gel (2% w/v) and then validated by melting curve analysis. The 2^-ΔΔCt^ method was used to calculate the relative gene expression. Primers used for real-time PCR analyses were designed using Primer 3 (http://frodo.wi.mit.edu/).

### Subcellular localization and BiFC assay

Because of HbFd1 with the chloroplast transit peptide (cTP), HbFd1 was fused to the N-terminus of GFP. To construct the HbFd1::GFP fusion protein, *HbFd1* cDNA containing *HindIII* and *SmaI* restriction sites without stop codon was amplified by RT-PCR using the primers (S1 Table). The amplified cDNA fragment was fused to GFP and cloned into the pGreen0029-35S binary vector. Then HbFd1::GFP construct was transformed into *Arabidopsis* protoplast as described by Abel & Theologis [17] using single GFP as positive control and empty vector as negative control. After transformation protoplast cells were incubated at 23°C in the dark overnight.

BiFC assay was performed according to Waddt et al. [18]. For generation of the BiFC vectors, the coding regions of *HbCIPK2* and *HbFd1* were subcloned via *BamHI*/*KpnI* and *BamHI*/*EcoRI* into pUC-SPYNE and pUC-SPYCE (provided by the laboratory of Jorg Kudla in Schlossplatz, Germany), respectively. The constructs of SPYNE::HbCIPK2/HbFd1::SPYCE were co-transformed in *Arabidopsis* protoplasts which were isolated from 4-week-old wild-type plants together with AtCBF1::RFP as reference control (fusion vector AtCBF1::RFP were constructed by Li et al. [15]). After incubation at 23°C in the dark for 12–18 hr, YFP signal was imaged using a Nikon inverted fluorescence microscope TE2000-E equipped with a D-Eclipse A1 spectral confocal laser scanning system (Nikon, Tokyo, Japan).

GFP and YFP fluorescence was observed at the emission of 488 nm, RFP at 543 nm, and auto-fluorescence of the chloroplast at 640 nm.

### Immunoprecipitation and western blot

In order to enhance the expression of target genes in the human embryonic kidney cell line HEK293T, an excellent heterologous expression system, kozak and tag myc sequences were amplified and fused to the N-terminus and C-terminus of HbCIPK2 using specific primers, respectively. Meantime, kozak and tag flag sequences were also amplified and fused to the N-terminus and C-terminus of HbFd1 without cTP using specific primers (S1 Table), respectively. We obtained HbFd1△cTP (mature HbFd1). After sequencing *HbCIPK2* and *HbFd1△cTP* fusion sequences were subcloned into vector pcDNA3.1(+) (Invitrogen) via *BamHI/EcoRI*, respectively. The above-mentioned vectors were named as pHbCIPK2-Myc and pHbFd1△cTP-Flag.

HEK293T cells were maintained and transiently transfected as described previously [19]. Cells grown in 60-mm peri-dishes were transfected with the indicated plasmids, the cells were harvested and lysed in ice-cold lysis buffer [150 mM NaCl, 50 mM Tris (pH 7.6), 1% NP40, 10% (v/v) glycerol, and containing protease inhibitor mixture]. After centrifugation at maximum speed for 10 min, supernatants were collected and protein concentrations were measured by using the Bio-Rad protein assay kit. Equal amounts of proteins were used for immunoprecipitations and incubated with 1 μg of mouse monoclonal anti-Flag M5 antibody (1:2000, Sigma) or antibodies against c-Myc (1:2000, Santa Cruz) for at least 3 h with gentle rotation at 4°C, and then added 40 μl of protein G/A agarose beads (GE Healthcare, Buckinghamshire, UK) for 1 h at 4°C. Beads were then washed with cell lysis buffer three times and the bound proteins were eluted with 2× loading sample buffer and subjected to SDS-PAGE in 12% polyacrylamide gels, followed by blotting onto nitrocellulose filter membrane with the corresponding antibody. Immunoreactive bands were detected using SuperSignal Chemiluminescence (Thermo Scientific Pierce) and exposed to X-ray film (Kodak X-OMAT BT).

### Investigation of the interaction segment by yeast-two hybrid assay

The vectors were first constructed for identification of the interaction segment using yeast-two hybrid assay. *HbCIPK2* cDNA containing *SalI* and *SpeI* restriction sites was amplified by RT-PCR using the primers (S1 Table), after sequencing *HbCIPK2* was inserted into vector pDBLeu (Invitrogen) via *SalI* and *SpeI*. For construction of *HbFd1* deletion vectors, reverse primers were designed at the No. 133 and 123 amino acid (total 143 amino acids), respectively. Two *HbFd1* deletion sequences were inserted into vector pPC86 (Invitrogen) via *SalI* and *EcoRI*, respectively, using wild-type *HbFd1* as reference. At last vector HbCIPK2△C1 contained less 10 amino acids than wild-type, HbCIPK2△C2 represents deletion of 20 amino acids. The indicated vectors were transformed into yeast cells and identified as described by method above.

## Results

### Yeast two-hybrid library screen

To identify HbCIPK2-interacting proteins, we utilized yeast two-hybrid screens with HbCIPK2 as bait and a *H*. *brevisubulatum* cDNA library as prey. The cDNA library was constructed from the bulked seedlings stressed by salt and drought. *HbCIPK2* was constructed into bait vector by gateway technology according to instructions, 35 mM 3-amino-1,2,4-triazole has been used to monitor the expression of the reporter *HIS*.

In total, 14 prey clones were identified by our yeast two-hybrid library screens, which were capable of supporting yeast growth when co-expressed with HbCIPK2. After sequencing, database searches were performed using the BLAST Net-work Service (NCBI, National Center for Biotechnology Service) (http://www.ncbi.nlm.nih.gov/BLAST). Eventually, 14 clones with high confidence scores represented unique putative HbCIPK2-interacting partners (Table 1 and S1 Text). The identified proteins are involved in variety of biochemical pathways, including photosynthesis, sugar metabolism and ion transport.

**Table 1:** Yeast two-hybrid library screen hits and scores.

Clone ID	Putative function	Identified time	Length (bp)	Reference organism	e-Value
Classification				accession no.	
20120406–26			510	*Hordeum vulgare*	
Transporter/Ion	Putative Magnesium transporter MRS2	1	Partial	AK370290	0.0
20120720–193			440	*Brachypodium distachyon*	
Transporter/Ion	Putative zinc transporter	1	Partial	XM_010230980	7e-165
20120813–48			590	*Brachypodium distachyon*	
Signal transduction	Receptor-like protein kinase	1	Partial	XM_003558884	5e-145
20120720–230			980	*Brachypodium distachyon*	
Metabolism/energy	ATP-dependent zinc metalloprotease FTSH 5	1	Partial	XM_003569192	0.0
20120720–66			430	*Triticum aestivum*	
Metabolism/enzyme	Cyclophilin A-1 (CyP1)	2	Partial	AF262982	1e-125
20120406–28			420	*Brachypodium distachyon*	
Metabolism/enzyme	4-coumarate-CoA ligase-like 4	3	Partial	XM_010230943	2e-130
20120720–281			1317	*Triticum aestivum*	
Metabolism/sugar	Fructan 6-exohydrolase	5	Partial	XM_006660402	0.0
20120726–37			426	*Brachypodium distachyon*	
	RNA polymerase II C-terminal domain phosphatase-like 1	1			3e-124
Metabolism/protein			Partial	XM_010241867	
20120726–69			540	*Brachypodium distachyon*	
Metabolism/protein	Desumoylating isopeptidase 2-like	1	Partial	XM_003574201	5e-154
20120726–77			320	*Brachypodium distachyon*	
Metabolism/protein	Mitochondrial phosphate carrier protein 3	1	Partial	XM_003570512	5e-68
20120813–160			432	*Triticum aestivum*	
Metabolism/protein	*PetF* gene for ferredoxin	3	CDS	X75089	0.0
20120813–89			600	*Brachypodium distachyon*	
Metabolism/protein	60S ribosomal protein L18-2-like	1	CDS	XM_010230343	0.0
20120813–144			420	*Hordeum vulgare*	
Function unknown	Uncharacterized protein	1	Partial	AK365792	2e-144
20120726–105			650	*Hordeum vulgare*	
Function unknown	Unknown protein	1	Partial	AK362967	2e-164

To validate the results obtained from the yeast two-hybrid library screening, individual identified clone was transformed together with HbCIPK2 bait vector into an independent yeast strain. Pairwise two-hybrid assay indicated that the library screen was positive.

### *HbFd1* encodes leaf-type ferredoxin with cTP

The clone 20120813–160 with intact CDS was used to further study. Gene sequence analysis revealed that it belongs to *Fd* gene family, and its full-length cDNA is 786 nt long, with an open read frame (ORF) of 432 nt that encodes a ferredoxin protein of 143 amino acids with cTP of 39 amino acids (S1 Fig). Blast search showed the higher similarity of its coding sequence with plant ferredoxins (Fds). Especially it is closest to barley Fd1 (HvFd1), then to rice Fd1 (OsFd1), showing 80% and 73.5% sequence identity, respectively. Hence this gene is designated as *HbFd1*, a novel member of plant *Fd* gene family.

**Fig 1:**
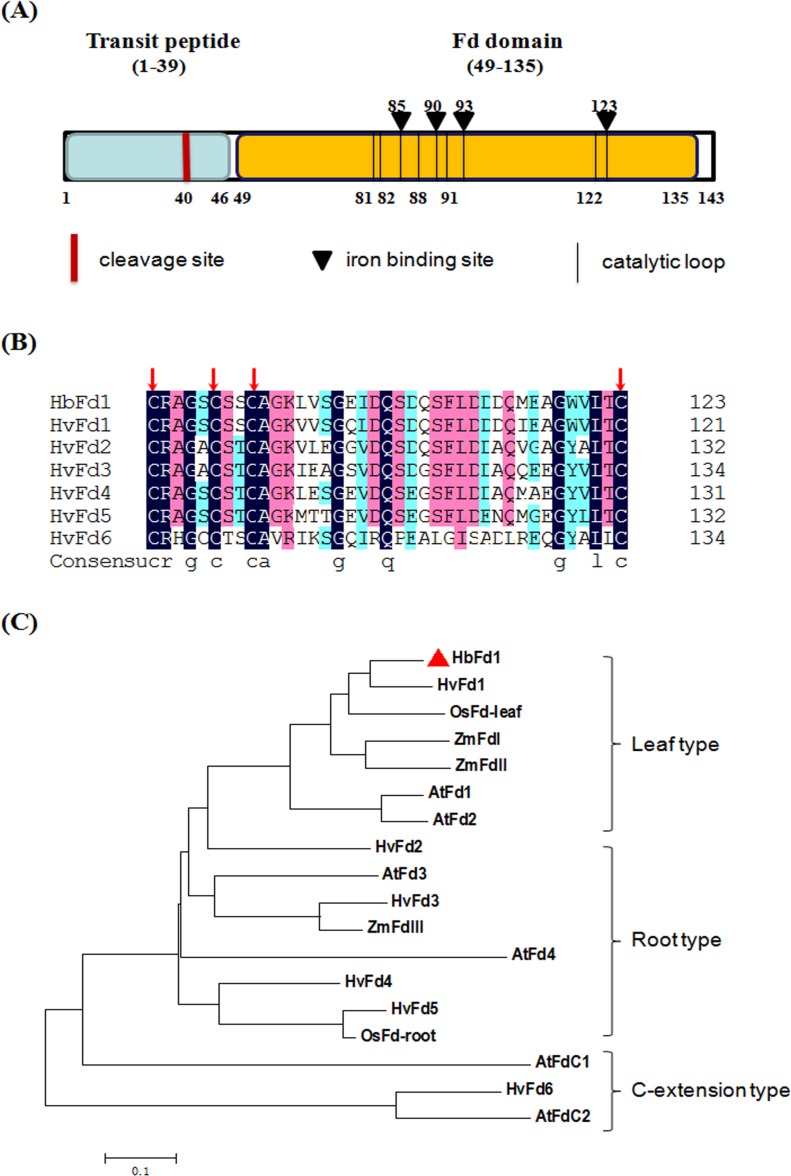
Schematic diagram, alignment and phylogenetic tree of HbFd1. (A) Schematic diagram of HbFd1. Chloroplast transit peptide sequence and cleavage site were depicted and predicted by the ChloroP1.1 server (http://www.cbs.dtu.dk/services/ChloroP/). The iron binding sites and catalytic loop of HbFd1 were predicted by NCBI Conserved Domian Search (http://www.ncbi.nlm.nih.gov/Structure/cdd/wrpsb.cgi). (B) Peptide alignment of active center of HbFd1 with that of six barley Fd proteins using DNAman software. Different colored shadings indicate identical extent of residues. Four highly conserved Cys residues are marked with red arrow. Sequences used: HvFd1 (AK253061), HvFd2 (AK250621), HvFd3 (AK358881), HvFd4 (AK371693), HvFd5 (AK252277) and HvFd6 (AK367145). (C) Phylogenetic analysis of HbFd1. The tree was generated with amino acid sequences of HbFd1 and some identified Fd proteins from rice, barley, maize and *Arabidopsis* using MEGA4, with 1000 bootstrap replications. The sub-groups of Fd proteins were depicted. Accession numbers for proteins are, rice leaf (BAA06436), rice root (BAA06456), maize I (P27787), maize II (O80429), maize III (P27788), *Arabidopsis* AtFd1 (At1g10960), AtFd2 (At1g60950), AtFd3 (At2g27510), AtFd4 (At5g10000), and AtFdC1 (At1g32550) and AtFdC2 (At4g14890).

To further characterize HbFd1, A search using the ChloroP 1.1 server (http://www.cbs.dtu.dk/services/ChloroP/) and the BLAST Net-work Service (NCBI, National Center for Biotechnology Service) (http://www.ncbi.nlm.nih.gov/Structure/cdd/wrpsb.cgi) revealed the deduced HbFd1 contained Fd domain which was located between amino acid residues 49 and 135, and there were four iron binding sites and several catalytic loops in this domain. The cTP was predicted by the ChloroP server, showing that cTP was located between amino acid residues 1 and 39, of which a potential cTP cleavage site was predicted at the residue 40 (Fig 1A). The structure indicated that HbFd1 was grouped into chloroplast Fds, and the characteristic of HbFd1 may determine its subcellular localization and functions.

Barley (*H*. *vulgare*) is the most relative species for wild barley (*H*. *brevisubulatum*), six HvFds have been identified by database search. Multiple alignment analysis showed that HbFd1 has high homology with six HvFds in which there are four highly conserved cysteine (Cys) residues, these four sites also were called iron binding sites responsible for photosynthetic electron transport (Fig 1B). Four highly conserved Cys residues constitute active center in the 3D structure [7].

All the higher plant Fds are categorized into leaf-, root- and extended C-terminus type, and each type may be involved in different function [1]. Phylogenetic analysis of HbFd1 with representative Fd proteins from database indicated that HbFd1 is clustered into the leaf type subgroup which consists of barley HvFd1, rice OsFd leaf type, maize ZmFdI and II, and *Arabidopsis* AtFd1and II (Fig 1C). It suggested that HbFd1 may belong to typical photosynthetic Fd proteins and play a key role in photosynthetic metabolism.

To further confirm whether *HbFd1* has no intron in *H*. *brevisubulatum* genome, we amplified *HbFd1* gene from cDNA and genomic DNA. The sequencing result indicated that *HbFd1* is intron-less.

### HbFd1 mainly expressed in shoot and is localized in the chloroplast

**Fig 2:**
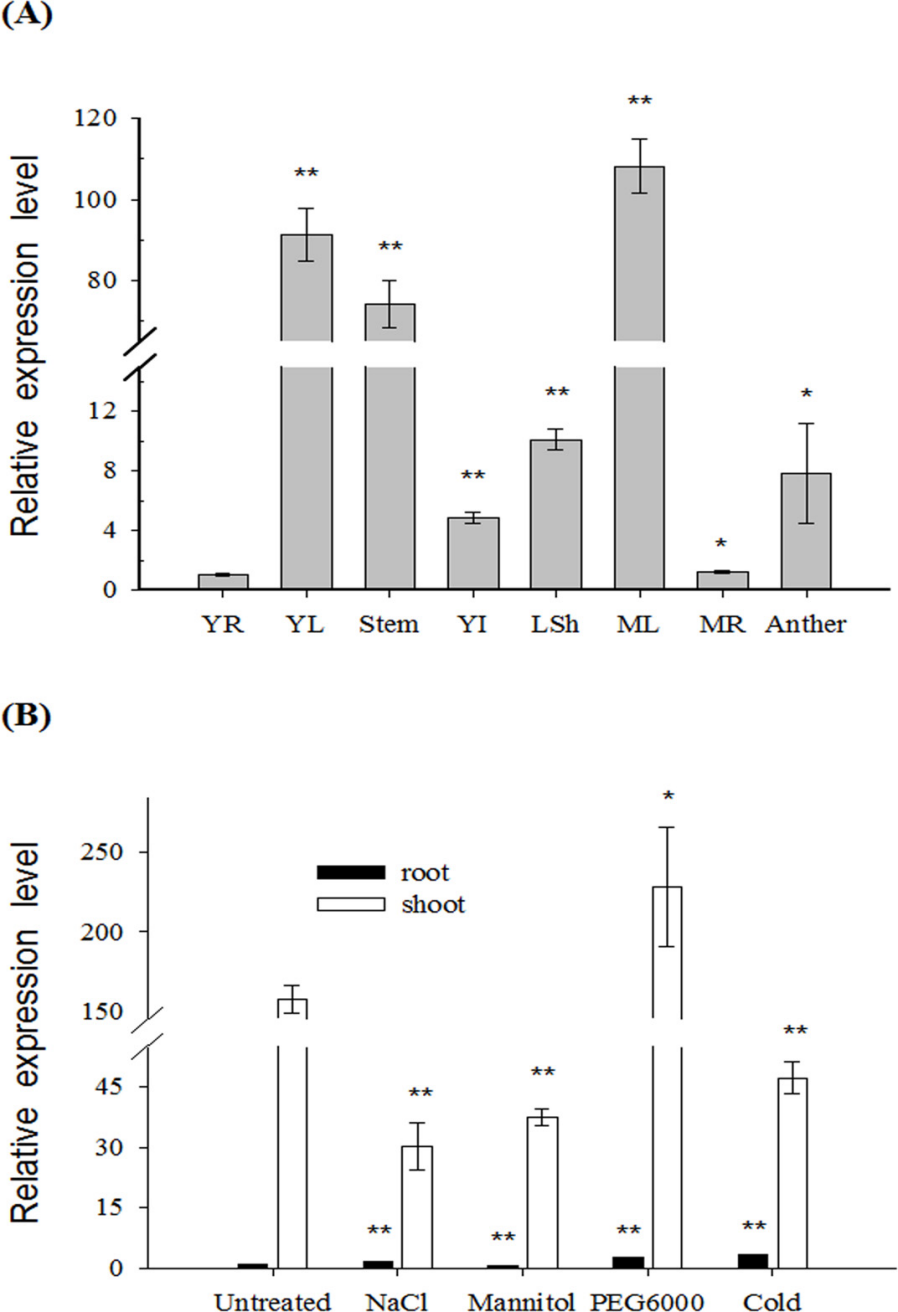
Expression analysis of *HbFd1* in *H*. *brevisubulatum* by real-time PCR. (A) Expression pattern of *HbFd1* from different tissues of *H*. *brevisubulatum*. YR, young root; YL, young leaf; steam; YI, young inflorescence; LSh, leaf sheath; ML, mature leaf; MR, mature root and anther. (B) Expression pattern of *HbFd1* in *H*. *brevisubulatum* under 350 mM NaCl, 350 mM mannitol and 10% PEG6000 stressed for 6 hrs, and 4°C for 12 hrs, respectively. All assays were performed in triplicate. Significant differences were determined relative to each control using a student’s t-test [*P*-values < 0.05 (*) and < 0.01 (**)]. Bars: SD.

To investigate the expression pattern of *HbFd1*, real-time PCR was used to monitor the spatial and temporal changes in different tissues and under abiotic stresses. Different tissues including (mature leaf, root and young leaf, root, stem, leaf sheath, inflorescence and anther) were used to investigate the expression of *HbFd1*. Interestingly, It mainly expressed in green tissues, with the level of expression reaching the maximum in mature leaves, and the mRNA of *HbFd1* in anther and young inflorescence was higher than that in roots (Fig 2A), confirming that *HbFd1* expression is shoot-specific. Furthermore, the expression of *HbFd1* was up-regulated by 10% PEG6000 whereas down-regulated by 350 mM NaCl, 350 mM mannitol and low temperature (4°C) for 6 hrs (Fig 2B).

**Fig 3:**
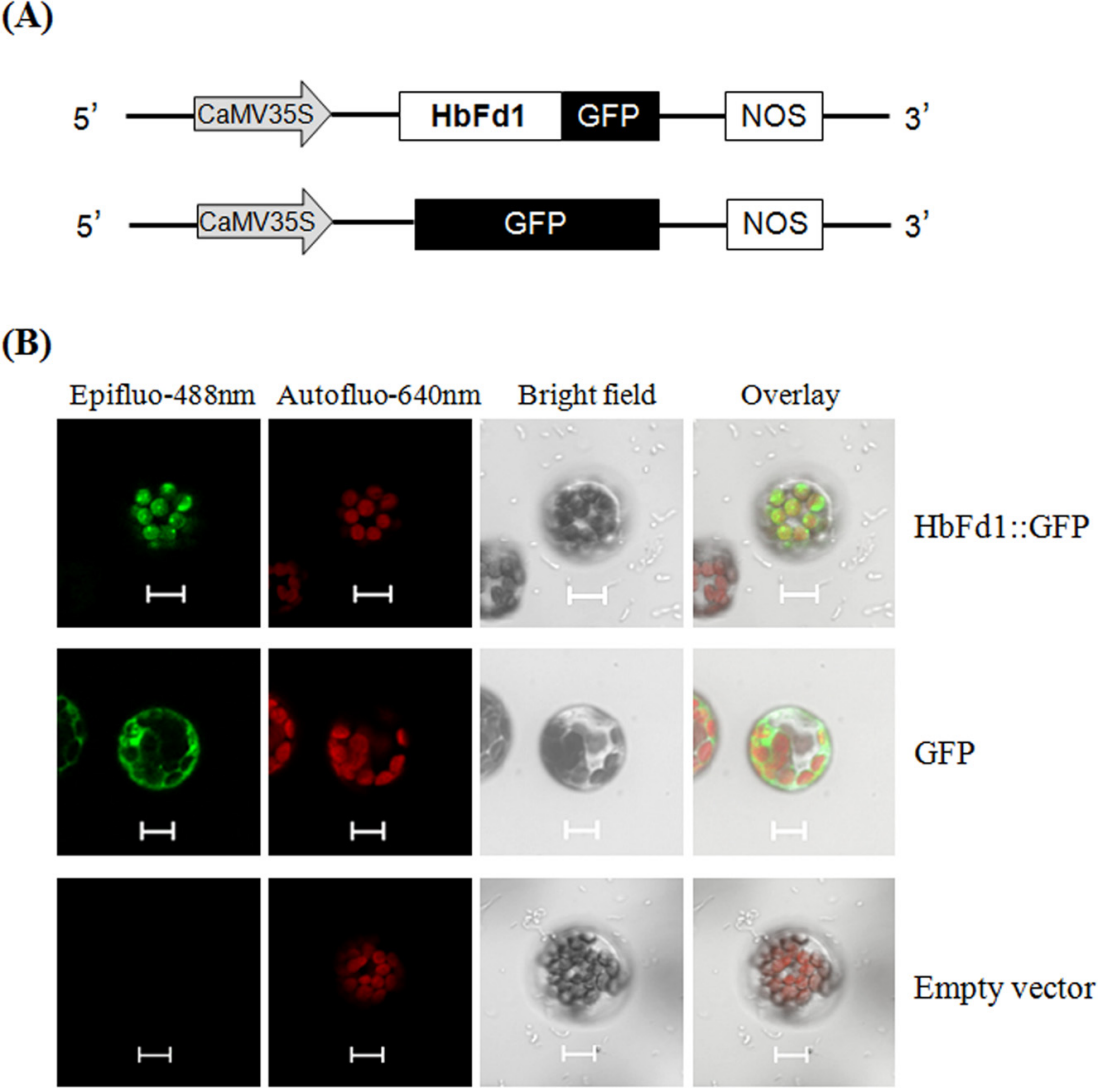
Subcellular localization of HbFd1 in *Arabidopsis* protoplast. (A) The vectors were used for subcellular localization. (B) Subcellular localization of HbFd1::GFP (top panel) with GFP as the positive control (middle panel) and empty vector as the negative control (bottom panel). At least twenty cells were observed, bars = 10um.

To examine the subcellular localization of HbFd1, HbFd1 was fused to the N-terminus of GFP due to the cTP in HbFd1, and transiently introduced into the *Arabidopsis* protoplast. The results showed that HbFd1 was localized to the chloroplast which showed red auto-fluorescence signal (Fig 3, top panel). In contrast to GFP alone, it was localized to the whole cell (Fig 3, middle panel). However, no fluorescent signal was observed expressing empty vector (Fig 3, bottom panel).

These results of expression pattern were consistent with bioinformatics analysis for HbFd1, suggesting that HbFd1 is a chloroplast Fd protein involved in photosynethic metabolism.

### HbFd1 interacted with HbCIPK2 in different levels

**Fig 4:**
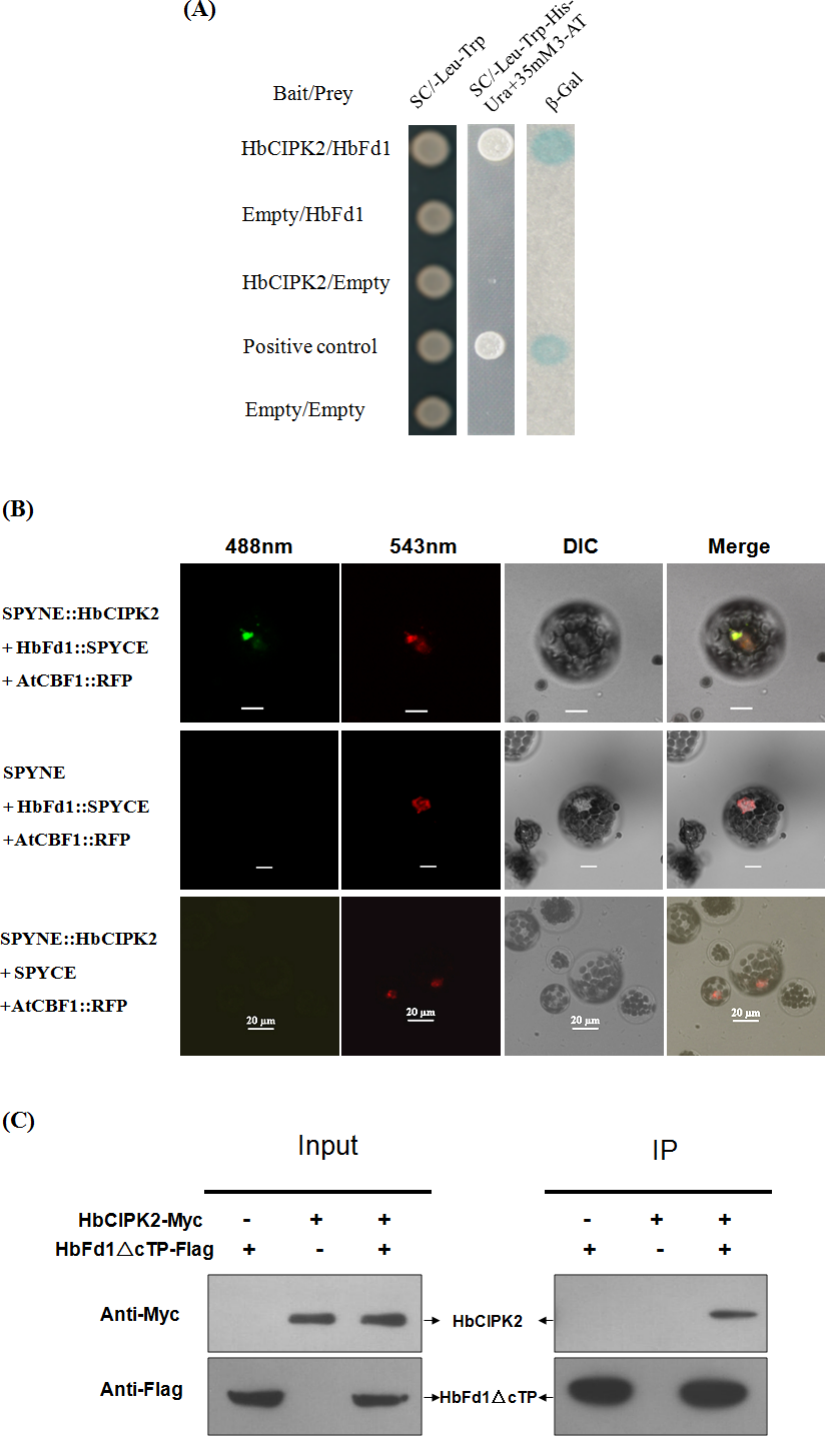
HbCIPK2 interacted with HbFd1. (A) Examination of the interaction between HbCIPK2 and HbFd1 in yeast. The interactions were verified by the yeast growing on selective medium (SC/-Leu-Trp-His-Ura with 35 mM 3-AT) and conducting ß-Gal assays. Bait vector with HbCIPK2 or prey vector with HbFd1 was transformed with empty vector as negative control, and the plasmids pPC97-Fos and pPC86-Jun provided by Invitrogen system were used as positive control. (B) Co-BiFC assay to verify the interaction of HbCIPK2 and HbFd1 in *Arabidopsis* protoplasts. Construct AtCBF1::RFP was used as reference to co-localize the interaction of HbFd1 with HbCIPK2, and transformants expressing SPYNE/HbFd1::SPYCE and SPYNE::HbCIPK2/SPYCE were used as negative controls. (C) Co-IP assay to show the interaction between HbCIPK2 and mature HbFd1 in cell line HEK293, co-expressing HbCIPK2-Myc and HbFd1△cTP-Flag. Cell proteins before (Input) and after (IP) immunoprecipitation were separated in SDS-PAGE gels, transferred onto the nitrocellulose membranes, and analyzed by protein gel blotting with antibodies as indicated. All assays repeated three times.

In order to avoid the influence of unknown sequence from clone 20120813–160 on the interaction between HbCIPK2 and HbFd1, the CDS region of *HbFd1* was amplified and constructed into the prey vector by gateway technology, the HbFd1 prey vector was transformed together with the HbCIPK2 bait into an independent yeast strain, and the prey or bait vector was transformed with empty vector as control, then screened on additional selective media. The pairwise interaction showed that HbFd1 interacted with HbCIPK2 in yeast strain (Fig 4A).

To confirm the interaction between HbCIPK2 and HbFd1 in vivo, co-BiFC assay was employed to test in cellular level using RFP fusion protein as reference. *AtCBF1* was used as reference gene, AtCBF1 has been proved to localize to the nucleus, so it was fused with *RFP* to monitor where the target proteins will co-express. Thinking of HbFd1 with cTP and cleavage site, HbFd1 was fused to split YFP at the N-terminus. *Arabidopsis* protoplast was transiently co-transformed with SPYNE and SPYCE fused constructs as well as *35S*:*AtCBF1-RFP*. Strong green fluorescence signals (green-color was selected to show the signal of YFP protein in this assay) were observed in cells only co-expressing SPYNE::HbCIPK2 and HbFd1::SPYCE combination (Fig 4B, top panel), which indicated reconstitution of the YFP protein as a result of interaction between HbCIPK2 and HbFd1. The green fluorescence signal was observed in the nucleus, which overlapped with the red fluorescence signal in the same cell expressing AtCBF1::RFP. However, expression of single fusion protein (SPYNE::HbCIPK2 or HbFd1::SPYCE) together with construct AtCBF1::RFP did not result in a green fluorescence signal while a red fluorescence signal still can be observed (Fig 4B, bottom two panels).

To verify this interaction is independent of plant system, we used the human embryonic kidney cell line HEK 293T, an excellent heterologous expression system, to identify the association between HbCIPK2 and HbFd1 in vitro. To correctly express the HbFd1 in HEK293 cells, we generated HbFd1 without cTP segment (HbFd1△cTP) which is putative mature HbFd1. Binding assays by co-immunoprecipitation and western blot analyses were conducted in mammalian cells. HEK293T cells were co-transfected with pHbCIPK2-Myc and pHbFd1△cTP -Flag or transfected with each plasmid individually. First we confirmed all of these plasmids can be expressed by western blot using appropriate antibodies (Fig 4C, left panel). Immunoprecipitation was performed with an anti-Myc antibody, then complex was run a SDS-PAGE, transferred to nitrocellulose membrane and western blotted with anti-Flag antibody or anti-Myc antibody, our data confirmed the interaction between HbCIPK2 and HbFd1 in vitro (Fig 4C, right panel).

Taken together, HbFd1 is one of exact interacting partners of HbCIPK2. Of these identified clones, *HbFd1* is a gene with possible function relevant for cellular metabolism. Hence, it was a preferred gene for further studies.

### HbCIPK2 may interact with the C-terminus of HbFd1

**Fig 5:**
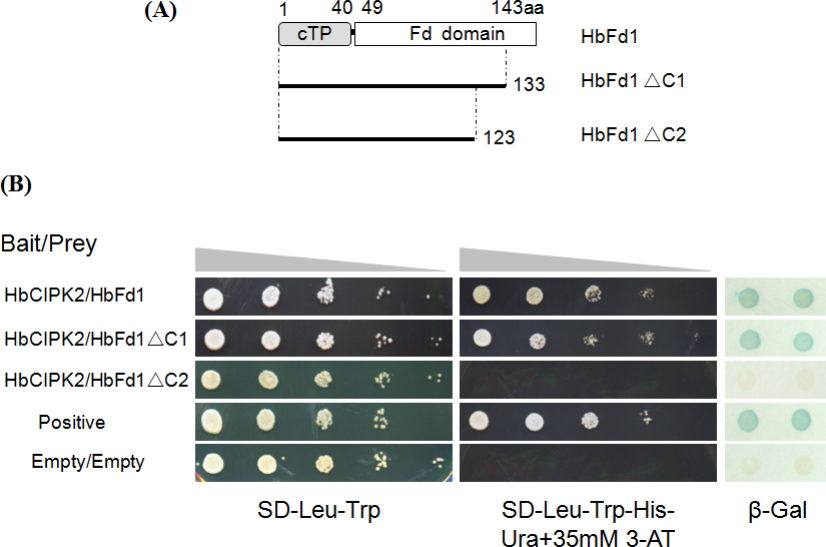
HbCIPK2 interacted with the C-terminus of HbFd1 in yeast. (A) Schematic diagram of mutant and wild type of HbFd1. HbFd1△C1 represents deletion of 10 amino acids in the C-terminus of wild-type HbFd1, and HbFd1△C2 means deletion of 20 amino acids in its C-terminus. (B) Investigation of the interaction between HbCIPK2 and mutant HbFd1. 10-fold dilutions (A_600_ of 1–10^−4^) of the yeast Mav203 strain harboring the bait vector and the indicated prey construct were spotted on selection media without Leu and Trp (selection for positive transformants) or without Leu, Trp, His and Ura (with 35 mM 3-amino-1,2,4-triazole; selection for interaction) and incubated for 3–5 days at 30°C. Meantime, the interaction was confirmed by ß-Gal assays. The growth of cells transformed with wild-type HbFd1 and HbCIPK2 was used as reference. The plasmids provided by Invitrogen system were used as positive and negative control to monitor the conditions in this assay.

To investigate which part of HbFd1 interacted with HbCIPK2, the mutant type of HbFd1 was first generated. According to the results of the research on crystal structure that plant Fd proteins have three flexible segments through which the protein-protein interactions can be achieved [7], the C-terminus of HbFd1 was deleted step by step. HbFd1△C1 contained less 10 amino acids than wild-type, HbFd1△C2 represents deletion of 20 amino acids (Fig 5A). The prey vector containing HbFd1△C1 or HbFd1△C2 was transformed with the HbCIPK2 bait vector into yeast cell using wild-type of HbFd1 as reference. The results indicated that the transformed yeast cells with HbFd1△C1 can grow as the wild-type in selected media, but the cell with HbFd1△C2 can’t grow (Fig 5B), suggesting that the segment between amino acid residues 123 and 133 in the C-terminus of HbFd1 may be involved in the interaction of HbFd1 with HbCIPK2.

## Discussion

### The interacting partners or sites of HbCIPK2 and HbFd1 were identified

It is vital to know the manner of protein-protein interactions for understanding biological events. In this study, to obtain HbCIPK2-interacting proteins we used HbCIPK2 as bait to screen *H*. *brevisubulatum* cDNA library. CIPKs are one kind of protein kinases involved in plant growth, development and response to abiotic stresses. To date, many reports focused on CIPK-mediated pathways in which CIPK interacts with CBL, then regulates downstream target proteins, especially responsive proteins to abiotic stresses such as AKT1, SOS1 as well as RBOHF and so on [9–11]. HbCIPK2 has been screened out by cDNA-AFLP technique from *H*. *brevisubulatum* which is an obligate halophytic grass native to salinized Inner Mongolia prairie [15]. In previous study we have reported that HbCIPK2 conferred salt and drought tolerance, but which process HbCIPK2 may participate in deserves research. Even though yeast-two hybrid assay has limitations and can yield false positives and false negatives, the results from the assay will aid in highlighting some components of HbCIPK2 interactome.

In conclusion, 14 putative proteins have been identified to interact with HbCIPK2, some are involved in ion transport, some are related to photosynthetic metabolism, and others may participate in sugar metabolism. The number of identified proteins is not only related to the quality of library, but also due to the limitation of yeast-two hybrid screening assay. Furthermore, some transcripts expressing at low levels in the library might not be detected, and interactions that require folding/assembly of *H*. *brevisubulatum* proteins might not be efficient in yeast [6]. Thus some proteins such as CBLs which have been proven to interact with CIPKs by pairwise yeast-two hybrid can’t be found in the list. Proteins which express at higher level can be detected in the Table 1. In some extent these proteins were involved in cellular metabolism. At last, HbFd1 was used for further studies due to its one-electron transfer protein in a number of essential metabolic reactions in addition to photosynthetic metabolism.

Recently published papers on the interaction of Fd mainly focused on the specific interaction with Fd-dependent enzymes which are considered to be important for efficient electron transfer between the two proteins, for example, ferredoxin-NADPI reductase (FNR) (involved in carbon assimilation) [20], sulfite reductase (SiR) (involved in sulfur assimilation) [21], ferredoxin-thioredoxin reductase (FTR) (involved in redox regulation of enzymes) [1], lipoate synthase (LipA) (involved in fatty acid biosynthesis) [8], as well as nitrite reductase (involved in nitrogen assimilation) [22] and so on. However, there is no report on how to regulate the interaction of Fd with Fd-dependent enzymes. Although Peden et al. identified global ferredoxin interaction networks in *Chlamydomonas reinhardtii* using large scale yeast-two hybrid screening, interacting partners of six Fds were mainly downstream enzymes in Fd-linked pathways which may exist crosstalk [6]. So it is important to identify regulatory Fd-interacting partners which may be a key switch for overlapping activities of Fd in multiple metabolic pathways.

To date, several studies on structure or site mutation aimed to reveal interaction sites of Fds with Fd-dependent enzymes. Kameda et al. mapped protein-protein interaction sites in the plant-type ferredoxin by crystal structure [7]. It has been proven that Fd has three flexible regions through which the protein-protein interactions are achieved. Our work showed that the segment between amino acid residues 123 and 133 in the C-terminus of HbFd1, where acidic residues were clustered, determined the interaction of HbFd1 with HbCIPK2. Somewhat surprisingly, the residues that interacted with HbCIPK2 were localized on the putative third flexible region of HbFd1. It is likely that this flexible nature of the segments contributes to fine-tuning of the specific interaction.

### It is important for HbCIPK2 to interact with HbFd1

Our work describes a large scale screening and identification of a novel HbCIPK2-interacting HbFd1 from halophyte *H*. *brevisubulatum*. We confirmed the interaction between HbCIPK2 and HbFd1 using pairwise yeast-two hybrid, BiFC assay in vivo and CoIP in vitro, the interaction of HbCIPK2 with HbFd1 is emerging an important link between CBL-CIPK regulatory system and Fd-dependent metabolic pathways.

We found that the expression of *HbFd1* showed tissue-specific, mainly in shoot chloroplast, it belonged to leaf-type subgroup. Interestingly, *HbFd1* was down-regulated under 350 mM NaCl, 350 mM mannitol and 4°C stressed for 6 hrs, which is consistent with the situations in previous reports [5]. However, the expression of *HbFd1* was up-regulated by 10% PEG6000 stressed for 6 hrs, which is different from the general view that ferredoxin was involved in stress-dependent repression process. This result will highlight a clue for *HbFd1* responsive to short-termed osmotic stress, suggesting that *HbFd1* may function in osmotic stress.

CIPK is plant-specific protein kinase which specially interacts with CBL to form active complex and phosphorylates downstream target proteins [12]. Till now, several target proteins responsive to abiotic stresses have been reported to be activated through the interaction of CIPKs. However, there is no report on the interaction between CIPK and ferredoxin. The interaction between HbCIPK2 and HbFd1 may be a base for regulation of HbFd1-linked pathways. We proposed HbCIPK2 may phosphorylate HbFd1 to mediate the activities to transfer electron to downstream target proteins under environmental stimuli, and activated HbFd1 may be a switch for multiple Fd1-dependent pathways. Of course, some assays need to be generated to verify this regulation process. Whatever, we suggested the interaction of HbCIPK2 with HbFd1 play a vital in the connection of CBL-CIPK network and Fd-dependent metabolic pathways.

### Possible model of interaction between HbFd1 and HbCIPK2 in the nucleus

**Fig 6:**
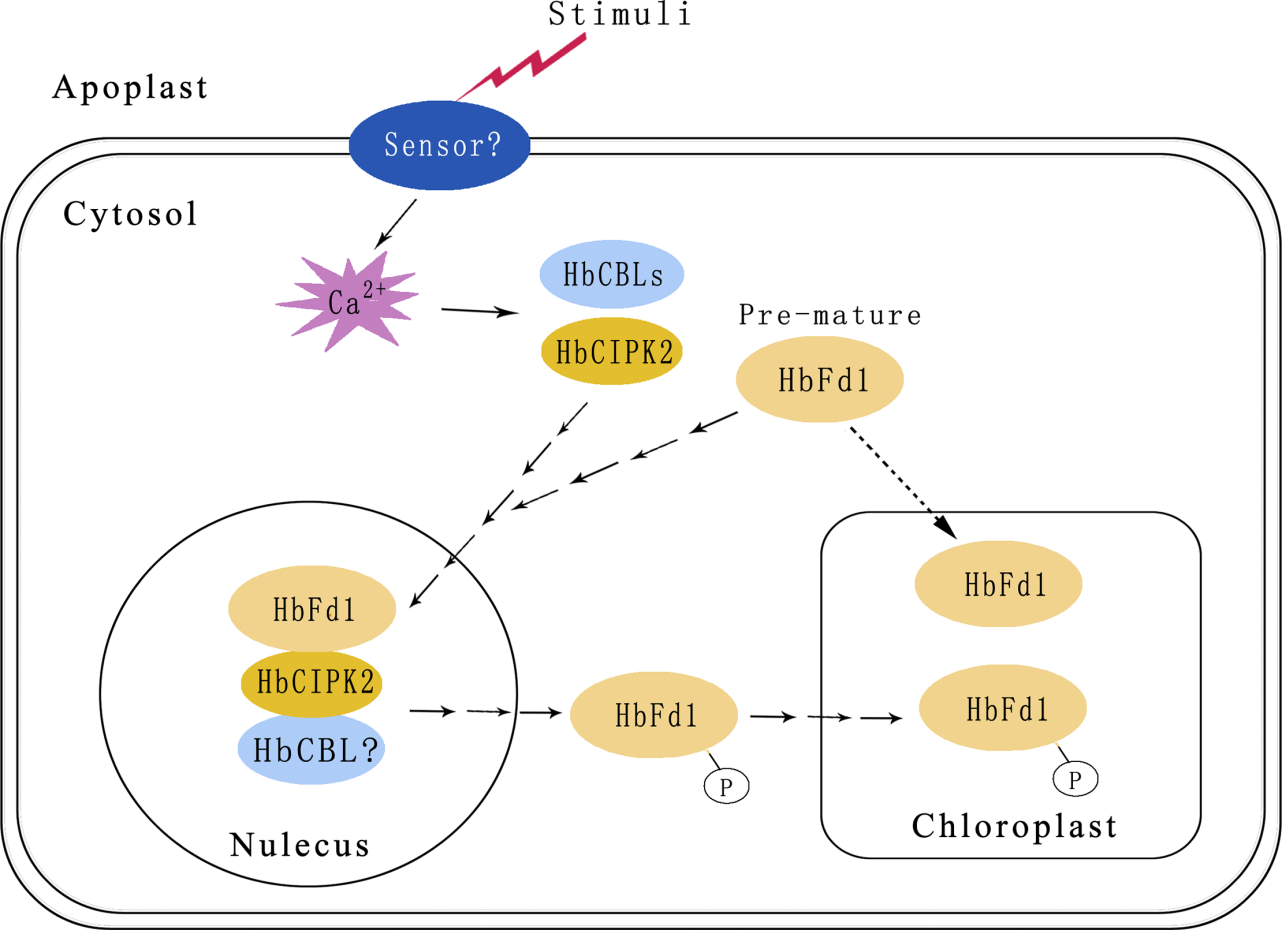
The proposed HbFd1 trafficking model.

The putative HbFd1 protein contains cTP predicted by the ChloroP server, and the subcellular localization assay also verified that HbFd1 expressed in the chloroplast after maturation. However, *HbFd1* is a kind of nucleic gene, not chloroplast gene, so transcription of *HbFd1* may perform in the nucleus, its translation may be done in the ribosome and at last HbFd1 may enter into the chloroplast to mature in normal condition. However, we inferred that HbCIPK2 complex may recruit pre-mature HbFd1 to the nucleus where HbCIPK2 interacted with HbFd1 under environmental stimuli, and HbFd1 may be modified by HbCIPK2 in the nucleus, after post-translation modification HbFd1 may enter into the chloroplast from the nucleus via trafficking system. Hence, we proposed that the model of HbFd1 trafficking from the nucleus to the chloroplast (Fig 6). The process may be just a puzzle, but this model may provide the highlight for the regulation mechanism of Fd protein by CIPKs.

## Supporting Information

S1 Fig Full-length cDNA of *HbFd1* and its putative amino acid sequence. Sequence of *HbFd1* from the prey vector identified by yeast-two hybrid screening, lowercase indicates cDNA sequence of *HbFd1* and uppercase indicates putative amino acid sequence. Full-length cDNA contains 3’ UTR (un-translation region) of 100bp and 254-bp 5’ UTR. Blue fonts indicate chloroplast transit peptide, red fonts show cleavage site of HbFd1 and purple fonts represent its Fd domain. Black triangles indicate iron binding sites in active center of HbFd1.
(TIF)

S1 Table Primers used in this work. (DOC)

S1 Text Clones and sequences identified in this work. (DOCX)
