# Supplementary figures and images for: Identification and Expression Analysis of a Novel HbCIPK2-Interacting Ferredoxin from Halophyte H. brevisubulatum
Source: PLoS One. 2015 Dec 4;10(12):e0144132. doi: 10.1371/journal.pone.0144132 (PMC4670114; doi:10.1371/journal.pone.0144132)

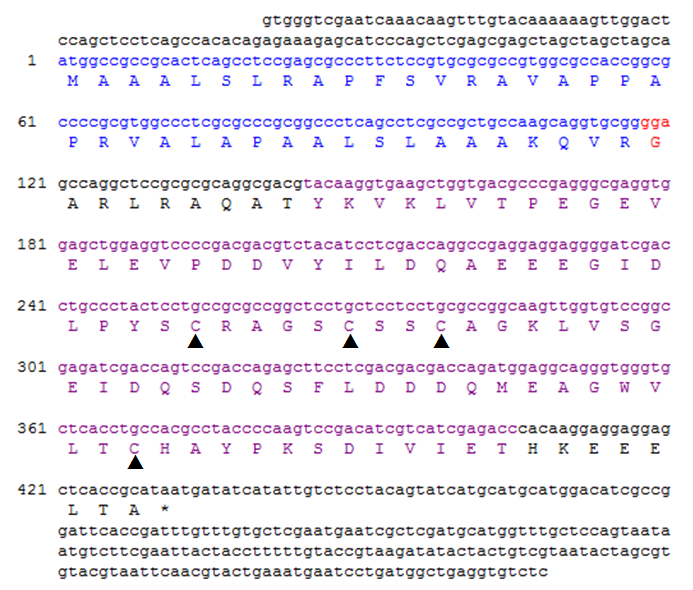

Supplement: S1 Fig — Sequence of HbFd1 from the prey vector identified by yeast-two hybrid screening, lowercase indicates cDNA sequence of HbFd1 and uppercase indicates putative amino acid sequence. Full-length cDNA contains 3’ UTR (un-translation region) of 100bp and 254-bp 5’ UTR. Blue fonts indicate chloroplast transit peptide, red fonts show cleavage site of HbFd1 and purple fonts represent its Fd domain. Black triangles indicate iron binding sites in active center of HbFd1. (TIF) [file pone.0144132.s001.tif]
