# Supplementary material for: Identification and Expression Analysis of a Novel HbCIPK2-Interacting Ferredoxin from Halophyte H. brevisubulatum
Source: PLoS One. 2015 Dec 4;10(12):e0144132. doi: 10.1371/journal.pone.0144132 (PMC4670114; doi:10.1371/journal.pone.0144132)
Supplement: S1 Table — (DOC) [file pone.0144132.s002.doc]

Supplemental Table S1

Primers used in the paper

| **Constructions** | **Restriction sites** | **Tag or other sequence** | **Primer sequence (5′-3′)** |
| --- | --- | --- | --- |
| **Primers for construction of pDEST32-HbCIPK2** | | | |
| HbCIPK2-attB1-F | *attB1* | TC | GGGGACAAGTTTGTACAAAAAAGCAGGCTTCATGGGGGAGCAGAAGGGGAA |
| HbCIPK2-attB2-R | *attB2* | C | GGGGACCACTTTGTACAAGAAAGCTGGGTCTCAACATGGTTGCTGCTGCGG |
| **Primers for BiFC vectors** | | |  |
| HbCIPK2-BamHI-F | *BamHI* |  | TGGATCCATGGGGGAGCAGAAG |
| HbCIPK2-KpnI-R | *KpnI* |  | TGGTACCTCAACATGGTTGCTGCTG |
| HbFd1-BamHI-F | *BamHI* |  | CGGGATCCATGGCCGCCG |
| HbFd1 -EcoRI-R | *EcoRI* |  | TGAATTCTTATGCGGTGAGCTCCTC |
| AtCBF1-BamHI-F | *BamHI* |  | TGGATCCATGAACTCATTTTCAGCTTTTTCTG |
| AtCBF1-KpnI-R | *KpnI* |  | TGGTACCGTAACTCCAAAGCGACAC |
| **Primers for CoIP vectors** | | |  |
| HbCIPK2-BamHI-F | *BamHI* | kozak | CGGGATCCGCCACCATGGGGGAGC |
| HbCIPK2-EcoRI-R | *EcoRI* | myc | GGAATTCTTACAGGTCCTCCTCTGAGATCAGCTTCTGCATTGATGCCATACATGGTTGC |
| HbFd1-delcTP-BamHI-F | *BamHI* | kozak | CGGGATCCGCCACCGCGACGTACAAGG |
| HbFd1-flag -EcoRI-R | *EcoRI* | flag | GGAATTCTTACTTGTCATCGTCTTTGTAGTCCATTGCGGTGAGC |
| **Primers for amplification of HbFd1 from cDNA and DNA** | | | |
| HbFd1-F |  |  | ATGGCCGCCGCACTCAGCCTC |
| HbFd1-R |  |  | TGCGGTGAGCTCCTCCTCCTTGT |
| **Primers for HbFd1 subcellular localization** | | | |
| HbFd1-HindIII-F | *HindIII* |  | AAAGCTTATGGCCGCCGCACTCAGCCTCCGA |
| HbFd1-SmaI-R | *Sma*I |  | TCCCGGGTGCGGTGAGCTCCTCCTCCTTGTG |
| **Primers for transcript of HbFd1 by real-time PCR** | | | |
| HbFd1-RT-F |  |  | GTCATCGAGACCCACAAGGAG |
| HbFd1-RT-R |  |  | TGCATGCATGATACTGTAGGAG |
| GAPDH-RT-F |  |  | TTTCGGAAGGATCGGGAG |
| GAPDH-RT-R |  |  | ACAGCCTTGGCAGCACCA |
| **Primers for identification of interaction site by Yeast-two hybrid** | | | |
| HbCIPK2-SalI-F | *SalI* | C | AGTCGACCATGGGGGAGCAGAAGGGGAA |
| HbCIPK2-SpeI-R | *SpeI* |  | TACTAGTTCAACATGGTTGCTGCTG |
| HbFd1-SalI-F | *SalI* | C | TGTCGACCATGGCCGCCGCACTCAGCCTCCGA |
| HbFd1-△C1-EcoRI-R | *EcoRI* | stop codon | TGAATTCTTAGATGACGATGTCGGACTTGGG |
| HbFd1-△C2-EcoRI-R | *EcoRI* | stop codon | TGAATTCTTAGCAGGTGAGCACCCACCCTGC |
