# Supplementary material for: Identification and Expression Analysis of a Novel HbCIPK2-Interacting Ferredoxin from Halophyte H. brevisubulatum
Source: PLoS One. 2015 Dec 4;10(12):e0144132. doi: 10.1371/journal.pone.0144132 (PMC4670114; doi:10.1371/journal.pone.0144132)
Supplement: S1 Text — (DOCX) [file pone.0144132.s003.docx]

Sequences of clones identified by yeast two-hybrid library screens

>20120406-26 (510 bp)

ATGATGAAGATACCCCACCAAACCCAAAAAAAGAGGGTGGGTCGAATCAAACAAGTTTGTACAAAAAAGTTGGAATAGGAGCAAATCTGCCGAGGCTAAACTCCAGTTTTCGACGTAGTGTGAGCATTGCAACCAGTATATATTTGGATAACGATGTGGAAGACCTAGAGATGTTGCTTGAGGCCTACTTCATGCAGCTGGATGGAATTCGCAACAGAATTTTATCGGTCCGAGAGTATATTGATGACACAGAAGACTACGTCAACATTCAACTCGACAACCAGCGAAATGAACTGATCCAGCTTCAGCTTACGCTGACCATCGCATCGTTTGGCATAGCTATCAATACCTTCATAGTCGGGGCCTTTGCGATGAACATCCCATGCCATCTTTACGACATCACGGATGGCAGCTTCTTTTGGCCATTTGTCGGAGGTACCTCATCGGGCTGCTTTGTGATCTCCGTCGTTTTGTTAGGGTACGCCTGGTGGAAGAAGTTGCTTGGTCCCT

>20120406-28 (420 bp)

GACGGTGACACCCGACGGATGGCTCAAGACCGGTGACCTATGCTACATAGACGAGGATGGGTATCTCTTTGTGGTGGACCGTCTGAAAGAGTTGATCAAATACAAAGGCTATCAGGTGCCTCCGGCAGAGTTGGAAGCTCTTCTGCTGACCCATCCAGAGGTTTCCGATGTCGCTGTTATTCCGTTCCCGGACAGGGACGTCGGCCAGTTCCCGATGGCCTATGTCGTGAGGAAGAAAGGGAGCAACCTGTCAGGCCAGGAGGTGATGGAGTTTGTGGCGAAACAGGTAGCACCGTACAAGAAGGTCAGGAAGGTGGCATTCGTGACAGACATCCCCAAGAACGCGTCTGGCAAGATACTGCGGAAGGATCTTATCAAGCTCGCGACGTCCAAGCTGTGAAATGTCGACCGTTATCCTGC

>20120720-193 (440 bp)

AGTTGGAGCGGCACGCAAGGCATATGGTCTAGGCCGCTACATGGTTCTTCCAGTCTCCCTTCACGGCCTACCACGGGGTGCTGCTGTTGCTAGTTGTGTATATGGTGCCACTGATAGCTGGCGAGGAGCCTTGGCAGCTGCAGCTCTGACCGGGCTTGCAGGACCAAGCGCGGCCATCAGTGCAATCCTTGCGAAGATTGGCTATGATGGACTGGACTATTGGATGGTGATTGCATGCGGGGCTTTGATCCCTAGCTTTGGCCGTGTCTTCAGACGCTCGCTGCGGCTGGACTTGAGGAAGAGTGTCTGCGGGCTCCTGATAGGTTTTGGTTTTGCTTGGGTGTGCTTGATGTCCACCAGATTCATCTGCTTGCACACCCCTTACTGCAATTCAGCTCCAGAAGCGGTCACATGATGTGTTCCTGTCAGATAGTCACATG

>20120720-230 (980 bp)

TCCTCGTGTTGATGCAGCAGTGTAGCGTATTCTTCCTGGGGGATACTCATCAGCTGACAGTTTGACGGAACAACCTGCTAATTTGGTGAATGAATATACACAATGCAAACCTGTGGATGCTAGGACCCGATACCAGCAACTCCCTTGGCCTGCGCTGCCGCTTGTTGTGCTGCTGCCGCGGCCGCAGCTGCCGCCGCCGCCGCTGGGGATGGGGGTGAAGCTGGCGTCTTCTGTGGGGCTTCAACAGCATGCCCCTGCTGCGGCTGTTTGTTATCTACTTGGGCCAACAAGTTCTTTATCTGGGTTCCGCTCATGGTCTCATGCTCAAGAAGTGCATTGGCTAGAGCATGGAGCTCCTTGTTGTGTTTGGTGAGGATAGTCTTTGCATTGTTGTATGCTCTCTCCAAGAAATTCTTCACCTCCTCCTCAATTAGAAGCCTCGTTTCTGAGCTCATAGTCTTTCCGTCATCTTCATAGTTGTACGAAACAAGACCCACCTGCTTGCTCATACCATACTGAGTAACCATTGCTCTTGCCATCAAAGTTGCTTGCTCAAAGTCGGATGAAGCACCAGATGTCACCTCGCTATCCCCAAATATAAGCTCCTCCGCCACTCGCCCTCCCATGCACACATCCAATCTTGCTAGCATCTGTTTCCTTGACACACTTGTCTCATCTTTGTCAGGGAGTTGAGCCACCATCCCAAGGGCCATCCCCCTGGGCACGATGGTAGCTTTGTGGACAGGGTGCGCACCATCCGTGTGCATGGCAACAAGGGCATGCCCTCCTTCATGGTATGCTGTCAGCTTCCTGCACTCATCAGAGATAACTGCTGACTTGCGCTCACTGCCCATCATGATCCGGTCCTTGGCATACTCCAGATCATTCATGCTAACGGATTTTGCCCCATCCATGGCAGCCCTAAGAGCAGCCACATTCACCAAGTTAGCAAGGTCAGCACCTGAAAATCCTCCAACTTT

>20120720-281 (1317 bp)

GCGGAGGACGAGGACGACTGCCGGAGCTGGCGCCGCCTCGACTACGGCCACGTGTACGCGTCCAAGTCCTTCTTCGACCCCAGCAAGAACCGGCGCGTGCTCTGGGGCTGGGCTAACGAGTCCGACAGCCTGGCCGACGACCTCGTCAGGGGATGGTCCGGTGTTCAGACTGTCCCGAGGAAGATATGGTTGGACGAAGACGGCAAGCAGCTGCGACAGTGGCCGATCGAGGAGATCGAGACGCTGAGGAGCAAGCGAGTGAACCTGCTGATACCGGAGGTGAACGCCGGCGGCGTCAACGAGATCATCGGCATCGTGGGCGAGCAGGCGGACGTGGAGGTCGTGTTCCAGATCCCGGCCCTGGAGCACGCCGACGTCCTGGAGCCCAACTGGCTGCTGAACCCGCAGAGCCTGTGCGGCGAGAAGGGCGCGTCCGTGCGGGGCAGCGTCGGCCCGTTCGGGCTGCTCGTCATGGCCTCCGGCGACATGCAGGAGCACACCGCCGTCTTCTTCAGGGTGTTCAGGCAGAACGACAAGTACAAGGTCCTCATGTGCACCGACCTCTCAAAGTCGAGCACCAGAGACGGCGTGTACAAGCCGCCGTACGGGGCCTTCGTGGACATGGACATCGAGGCGCAAGGGGGCAGCATCTCGCTCAGAACACTGGTTGACCACTCGGTGGTGGAGAGCTTCGGCGGCGGCGGCCGGACGTGCATCACGGCGCGGGTGTACCCGGACCACGTCGTGAACGGGAACAGCCACCTGTACGTGTTCAACAACGGCACCGGCGCCGTCAAGGTGTCCAGCCTCGACGCGTGGGAGATGGCCACGGCGACCGTGAACGTCCTCCCCGACGGGCTGATCGCGGCGAGCTCGGTGAGCAGAGCTCAAGCCTATTAGGATTCAGGAGCTGGGTTTTCGCTCGGTGGATGGATGGATGGATGGCCATATTGTGAATTTCTACGGCCGATTGGATTTGCCTGCCCAATTGTGGCTCAGGTGTACAGATGGAGGCTCCCTCATTGTTTGCCGGAACAGCACAGAACAAGGATTCTAGGATGCTGCGAAGGAAAGAAGATTGTAGTAGGAAGATTTGTGTCAGGTGTTAAGTTTTTTATACTGTTATAATAATACTAGTACTGTAGTATAGAAGTAGCACGCATGCCCTGCATGTTGGGTATTTGGCAACCATTAGAGTGTATTTCCGAGGTGAAAACGGGTGTCATGTAAAAGTATGATAGCCGTTGAGTTGTAAAAAATATTACTATTCAAATCTAAAAGGTATAAATTGTGATTAAAAAAAAAAAAAAAAAAAAA

>20120726-37 (470 bp)

TGCAAGCACGTCGGATGGGTCTAGATATATGCACGAGAGAGTTGATAACTCAACCAAGTCTGCTGGTTCTGTTGCTGCTCTTAAGGAACTTTGTACAGCTGAGGGGTATAACTTAATTTTCCAAGCTCAGCCATCTCCATTAGATAGTTTGATGGCAAGGAAAGAAGTTCATGCTCAGATTGAAATAGGTGGGCAAATTCTGGGAAAAGGAGTTGGAGCAACATGGGAGGAAGCTAAGATGCAGGCTGCTGATGGGGCTCTGGGAACTCTGAGATACATGCTTGGTCAACGTCCACTGAAACGGTCTGGATCTCCAAGGTCATTTGGATCCAATTATAATAAGCGATACAAGCCAGATTTCCAGCCGATGGTACAAAGGATTCCTTCTGGCAGATACTCTAGGAATGACAGTCGTGTTCCTTGATTGTTGTATCTGAATTCCGTGGGACACGATTCATCAGAAATTCATT

>20120726-66 (430 bp)

ATTGGCTCGACGGCAAGCACGTGGTCTTCGGCGAGGTCGTCGAGGGCATGGACGTCGTCAAGAACATCGAGAAGGTGGGCTCTCGCAGCGGCACCTGTGCCAAGCAGGTCGTCATCGCCGACTGCGGCCAGCTCTAGGTCCGTACTTCCGCCCGTCGCTCGCCGTCGTCGTCGTCGTCGTCGTCCTGCCGTGTGTCCCGTGTCGTGTCGTCGGTCTAAATAAGAATTGGCGTGAGTGTCGTGGTGTCGTGTCGTGTCTTCCCCTTGTCCGTCCGATGAGATCTCCCTGGTTGGTTTAGGAGGGTTTTAGGTGGACTTGCTGGATCTTGTGTCCGGCGGCCATGGTTATCTTCCCTTGTCTGCTGCTATCTTACCGTTTGAATGAATGAGATGAACTCCGCTATGAATTATCAAAAAAAAAAAAAAAAAAA

>20120726-69 (540 bp)

GGGGGACACTCCTACGACCTCCTCTCCAGGAACTGCAACCACTTCTGCGACGTGCTCTGCGACAGACTCGGCGTCCCCAAGCTTCCAGGCTGGGTTAATCGTTTTGCCAATGCTGGCGATACTGCTGTGGTGGTTGCTGAGAATACAGCAGTTAAGTTCAGGCAGGCTAAAACAGAAATTGTCAATGCTAGTAGAGTAGCATATAGATTCATGGCAGGCCTGACTTCGAAAAACCAGGCTTCACAAGAGGCCCCAGGTGACCAAAATAGAGGCGGCGGACCTACTTTCCAAGGAACATGGTTCAAGAATGTTGTTTCAGCTGGCGCAAAGCCGTCCACAAGCGGGTCAACTCCCTCACAAGAAGCTGACGATGCACCCCCCTTGCAGGGCCAGAAATCGGCAGAGCAGTCGACAAGGTTGTAGCCGTTGCTGTCACGGCAGACCTGCCGACCGTTGTATTGAAATCTGTAGTGTAACAGTAGCCAACATTTTCAAACATGTTTATTCACACATGAATCTATGTGCCCCTGCATTGCAACT

>20120726-77 (320 bp)

AAAAGTTGGAGACGATGACGATGTATTCCTGGTTGTGGTTATTGGTGGATGGGACTGTATCAGTTACTTGAGTTTAGCACCCGAAATCTTTTGTTATTGTAGCAGATGGTTATTGTGTCATCATTTTGATGAGAGGAACTCATTAAGCAGGCGAAATAGGCCTTTTTTGCCCCCCCGGGAAGTTGATGCTTGACATAATAAGTTTTAGGACAGCAAGGCTGTTAGAGCGCAGGTTCTGTCATCACCTGTACTGAGGGATTCCCATATAATCTTGTTTACCTCCAAATTTATGCTGTTGAAAAAAAAAAAAAAAAAAAAAA

>20120813-89 (680 bp)

TCCTCCTCCGCCGCCGCCGCCATGGGTATCGACCTCGTCGCCGGCGGCCGTAACAAGAGGACCAAGCGCACCGCGCCAAAGTCGGACGATGTCTACCTCAAGCTTCTGGTGAAGCTCTACCGCTTCCTGGTGAGGAGGACCAAGAGCCAGTTCAATGCCGTGATCCTGAAGCGCCTCTTCATGAGCAAGACCAACCGCCCGCCGCTCTCGCTTCGTCGCCTCGTCACGTTCATGGACGGCAAGGATAACCAAATCGCTGTGGTCGTGCGCACCATAACTGACGATAAGAGAGTTTACGAGGTGCCCGCGATCAAGGTGGCAGCGCTCAGGTTCACTGAGACGGCCAGGGCCCGCATTGTGAATGCCGGAGGAGAATGCCTCACCTTTGACCAGCTGGCGCTTCGTGCTCCTCTTGGCCAGAACACGGTTCTCCTGAGGGGTCCTAAGAATGCCCGTGAGGCAGTGAGGCACTTCGGTAAGGCTCCTGGTGTGCCTCACAGCCATACCAAGCCTTATGTCCGCTCTAAGGGAAGGAAGTTTGAGAAGGCAAGAGGAAGGAGGAACAGCAGAGGCTTCAAGGTCTAGGTTGTTGCATGGTGTTCATGTGCTCCACAGCTCGTGTTCGAGTCTGAGAGTTATGTTATTACTGAGTGCTAGGATCATCTAAATTTTGTTTGAAC

>20120813-89 (600 bp)

TCCTCCTCCGCCGCCGCCGCCATGGGTATCGACCTCGTCGCCGGCGGCCGTAACAAGAGGACCAAGCGCACCGCGCCAAAGTCGGACGATGTCTACCTCAAGCTTCTGGTGAAGCTCTACCGCTTCCTGGTGAGGAGGACCAAGAGCCAGTTCAATGCCGTGATCCTGAAGCGCCTCTTCATGAGCAAGACCAACCGCCCGCCGCTCTCGCTTCGTCGCCTCGTCACGTTCATGGACGGCAAGGATAACCAAATCGCTGTGGTCGTGCGCACCATAACTGACGATAAGAGAGTTTACGAGGTGCCCGCGATCAAGGTGGCAGCGCTCAGGTTCACTGAGACGGCCAGGGCCCGCATTGTGAATGCCGGAGGAGAATGCCTCACCTTTGACCAGCTGGCGCTTCGTGCTCCTCTTGGCCAGAACACGGTTCTCCTGAGGGGTCCTAAGAATGCCCGTGAGGCAGTGAGGCACTTCGGTAAGGCTCCTGGTGTGCCTCACAGCCATACCAAGCCTTATGTCCGCTCTAAGGGAAGGAAGTTTGAGAAGGCAAGAGGAAGGAGGAACAGCAGAGGCTTCAAGGTCTAGGTTGTTGCATGGTGT

>20120813-48 (590 bp)

AATTTTGATATTTCCAGCACCACTGGATACTTGGCCTATCCGTATTACATCGACTATGTTGTGGACGTTGAGGATGAAGGGATGCTGAAACTGGCCATTGGTGGCTCCAAGATGAGCCGACCTGGAGAAGCGAGCGGTTTCCTGAATGGGCTCGAGATAATGAGGATGAACAAAACGGGTGGCGGCATGGATGGGGATTTCCCTGTTGTTCTGGACATGGGGTACCTGGTCAGTAAGGGGTTTGGGGAATTCGCTCGCTCACTGCTGTGTGGTTTAGTCTTCGCCGGGCTGTTTTTGATGTTGGTCATGCTGGTGCTGAGGTTGAGGACCGAGCTGAAAAATAATGGCACGCTTTGGTTCAGCCAGTCAATGGATTCTGGTGAGGGGAAGTTGGCTAAAGCATATCAGCTTGTGCCCACCAAGGCAGACTATTGAGCAGCATGAGATTTGAGAAGCTTCAAATTGTGTCTCTTATCTGTATCTGATGACCTAATCTTGTGCTTCGGTTGTAAGTATCGTAGTTTACTAGTTTTACATTTCTACGCCTTTCATGGTTGAAGTTTCAATTGTTTGAGCTTGGCCAGTGCAAA

>20120813-144 (420 bp)

CACGCAAGCAAACAATGGCCGCCGCTCCATCCCCCGCCGTCGCCGCCGTCAGCAGCAGCGCGCCCTCGTCCGGGCTCCTGCCGCCGCGGCGCGGAGTGCCTTGCAGCGCCGTCCAGCTGCCGTCGATGAGGACAGCGGCGCTGGGTGGGGGCCGGATGGCGACGGTGGCGCGCGCGGTGGGCGACGTGGGCGCGGAGGGCAACACGTTCCTCATCGCCGGCGCCGTGGCCGTCGCGCTCGTCGGCACCGCCTTCCCCATCTTCTTCTCCCGCAAGGACACGTGCCCCGAGTGCGACGGCGCCGGGTTCGTGCGCAAGTCCGGCGCGACGCTGCGGGCGAACGCGGCGAGGAAGGACCAGGCGCAGATCGTCTGCGCCAACTGCAACGGCCTCGGCAAGCTCGGACAGATCGATAAGTAGA

>20120726-105 (650 bp)

GATGAAGCGATGCATCGTTCCTAGCATCCTGCTGATGCTTGCACTGCAGGCAGCCCTCCTCGTCGCCGGCGACGAGGTGGGCGCCATTCTCCTGCCGAGCCAAGGCCAAGCCATGGCGGCGGCCAAGAAGAGGCCGTGGAAGTGCTGCGACCAGGCGGTGTGCACCAGGTCCATCCCGCCGATCTGCACCTGCATGGACCAGGTCTTCGAGTGCCCCAAAACCTGCAAGGCCTGCGGGCCGTCCATGGCTGACCCGTCCCGCCAGGTCTGCCAAGACCAGTACGTCGGCGACCCCGGGCCCATCTGCCGGCCGTGGGAGTGCTGCGACCTGCCCCGGTGCACCAGGTCCAACCCGCCGACGTGCCAATGCCTGGACGAGGTCAAGCGGTGTGCTCCAACCTGCAAGACCTGCTTCCCGTCCAGGTCGCACCCGTCCCGCCGCGTCTGCAACGACCGCTACTTTGGGCCCTTCCCGCCCAAATGCACTCCGTCGGAGGCCGTTGCCGCCGGCGGCAACTAGCTACCACGCGCACGCGCGCGGCGCGCGTTGGTCTCCCGATCGACGCCGTCCAGTCTCCTCTTGCCCTGCAGCAGCAGCAGCAGCAGTGTGTAAGAGTAAAAATAAAAATGGATGGCCGGCCTCCGGCCAC
